# Genomic and molecular characterisation of a KPC-producing *Klebsiella pneumoniae* clinical isolate resistant to meropenem-vaborbactam, imipenem-relebactam, and ceftazidime-avibactam

**DOI:** 10.1186/s12863-026-01421-x

**Published:** 2026-05-09

**Authors:** Yu Wan, Joshua L. C. Wong, Julia Sanchez-Garrido, Wen Wen Low, Jane F. Turton, Fabio Morecchiato, Ilaria Baccani, Kirsty Dodgson, Gian Maria Rossolini, Neil Woodford, Gad Frankel, Elita Jauneikaite, Danièle Meunier, Katie L. Hopkins

**Affiliations:** 1 AMR & HCAI Division, UK Health Security Agency, London, UK; 2 NIHR Health Protection Research Unit in Healthcare Associated Infections and Antimicrobial Resistance, Department of Infectious Disease, Imperial College London, London, UK; 3 David Price Evans Global Health and Infectious Diseases Research Group, University of Liverpool, Liverpool, UK; 4 Department of Life Sciences, Imperial College London, London, UK; 5 Public Health Microbiology Reference Microbiology Division, UK Health Security Agency, London, UK; 6 Department of Experimental and Clinical Medicine, University of Florence, Florence, Italy; 7 Department of Medical Microbiology, Manchester University NHS Foundation Trust, Manchester, UK; 8 Careggi University Hospital, Florence, Italy

**Keywords:** *Klebsiella pneumoniae*, Antimicrobial resistance, Carbapenem resistance, β-Lactamase inhibitors, KPC-157, OmpK36, Insertion sequences, Hybrid genome assembly, Comparative genomics, Mutagenesis experiments

## Abstract

**Background:**

Resistance to carbapenems and third-generation cephalosporins is increasing in *Klebsiella pneumoniae* globally, restricting therapeutic options. The β-lactam/β-lactamase inhibitor combinations are widely used to circumvent β-lactamase-mediated resistance. In 2021, an unusual *K. pneumoniae* clinical isolate, KpMVR1, was recovered from a hospitalised patient in England, exhibiting resistance to meropenem-vaborbactam, imipenem-relebactam, and ceftazidime-avibactam. To investigate this phenomenon, we characterised the genome and antimicrobial susceptibility of KpMVR1 alongside two clonally related isolates susceptible to all three β-lactam/β-lactamase inhibitor combinations: KpMVS1, collected from the same patient 42 days earlier, and KpMVS2, from another patient in the same hospital.

**Methods:**

Illumina and MinION whole-genome sequencing were conducted for these three isolates, followed by hybrid genome assembly. Annotated genome assemblies were compared to identify genetic variation. Mutagenesis experiments were performed to verify predicted functional alterations.

**Results:**

All isolates belonged to clone ST8134 and carried *bla*_KPC-2_ alleles (KpMVR1: *bla*_KPC-157_; KpMVS1 and KpMVS2: *bla*_KPC-2_) in plasmids predicted to be conjugative. Insertion sequence IS*Ec68* caused a frameshift mutation in KpMVR1’s *ompK36* gene, reducing susceptibility to meropenem-vaborbactam and imipenem-relebactam. KPC-157 demonstrated decreased hydrolysis of imipenem and ceftazidime when compared with KPC-2. KpMVR1 also encoded a disrupted transcriptional repressor MarR and a destabilising mutation in AcrB, a component of the AcrAB-TolC multidrug efflux pump. An intact, iron-transporting *fec* operon was identified on a novel IncFII(pKP91)/IncFIB(K) plasmid unique to KpMVS2, possibly accounting for the cefiderocol resistance observed in this isolate.

**Conclusions:**

KpMVR1 carried multiple resistance-associated genetic alterations and likely developed its resistance profile through within-patient evolution. This study highlights the importance of routine screening for resistant pathogens in vulnerable patients to guide antimicrobial chemotherapy and the need to characterise underlying resistance mechanisms to assess the risk of onward dissemination.

**Supplementary Information:**

The online version contains supplementary material available at 10.1186/s12863-026-01421-x.

## Background

Meropenem and imipenem are broad-spectrum carbapenem antimicrobials parenterally administered to treat serious bacterial infections, such as those caused by Enterobacterales producing extended-spectrum β-lactamases (ESBLs) or AmpC-type cephalosporinases (AmpCs) [1–3]. Ceftazidime, a third-generation parenteral cephalosporin, is also widely used for treating severe bacterial infections, although it can be hydrolysed by ESBLs and AmpCs [4, 5]. In Gram-negative bacteria, carbapenems and cephalosporins diffuse through outer-membrane porins and enter the periplasm, where they inactivate penicillin-binding proteins, disrupting cell-wall synthesis with a bactericidal effect [6–8].

Combining β-lactams with β-lactamase inhibitors in antimicrobial chemotherapy is a widely employed strategy to circumvent β-lactamase-mediated resistance in bacteria. Vaborbactam, relebactam, and avibactam are non-β-lactam inhibitors of Ambler class A β-lactamases, such as ESBLs and *Klebsiella pneumoniae* carbapenemases (KPCs), as well as class C β-lactamases (AmpCs) [9]. Moreover, avibactam inhibits several class D β-lactamases such as OXA-48 and OXA-10 [10]. These inhibitors penetrate the outer membrane (OM) of Gram-negative bacteria via porins, blocking the active sites of β-lactamases in the periplasm [9, 11].

Meropenem-vaborbactam and imipenem-relebactam are recommended in the United Kingdom (UK) for treating adult patients (≥18 years of age) with severe multidrug-resistant bacterial infections where therapeutic options are limited, and ceftazidime-avibactam is recommended as an alternative when the disease-causing bacterium produces class D carbapenemase (e.g., OXA-48) [12–14]. Meropenem-vaborbactam resistance was not identified in *K. pneumoniae* clinical isolates across the UK as of 2020, while ceftazidime-avibactam resistance was identified in 6.1% of English *K. pneumoniae* isolates reported to the Second Generation Surveillance System (SGSS) of the UK Health Security Agency (UKHSA) between April 2019 and March 2020 [15]. Prevalence of imipenem-relebactam was below 1% in *K. pneumoniae* bloodstream isolates collected between 2015 and 2019 in the UK and Ireland [16]. Across the globe, resistance to these three agents has been documented in KPC-producing *K. pneumoniae* (KPC-*Kp*) in Italy (ceftazidime-avibactam: 6.5–12.9%; meropenem-vaborbactam: 3.2–14.5%; imipenem-relebactam: 1.6–4.3%; single-site from 2018 to 2020) [17], Greece (ceftazidime-avibactam and meropenem-vaborbactam: nil; imipenem-relebactam: 1.5%; single-site from 2017 to 2020) [18], and in multidrug-resistant *K. pneumoniae* in the USA (ceftazidime-avibactam: 2.2%; meropenem-vaborbactam: 2.9%; imipenem-relebactam: 5%; nationwide from 2018 to 2022) [19]. Notably, KPC-*Kp* in the ST512 clone exhibiting cross-resistance to all three agents caused an outbreak across three Italian hospitals in 2021 [20].

Identified mechanisms of resistance to meropenem-vaborbactam, imipenem-relebactam, and ceftazidime-avibactam include KPC overproduction [21, 22] and gain-of-function mutations in KPC [23, 24]. Disruption or transcriptional downregulation of *ompK35* (*ompF*) and *ompK36* (*ompC*), which encode non-selective porins that mediate the diffusion of β-lactams and β-lactamase inhibitors through the OM [25], has also been implicated [21, 23, 26–28]. Notably, the combination of OmpK36 inactivation, KPC-8 (KPC-3 variant V239G), and mutation of the transcriptional repressor gene *ramR*, has been shown to cause resistance to all three antimicrobial combinations [29, 30]. Similarly, meropenem-vaborbactam susceptibility of *K. pneumoniae* decreases when overexpression of the AcrAB-TolC multidrug efflux pump, which can result from the *ramR* mutation, is combined with inactivation of both *ompK35* and *ompK36* [9].

In 2021, KpMVR1, a KPC-*Kp* clinical isolate resistant to meropenem-vaborbactam, imipenem-relebactam, and ceftazidime-avibactam, was identified in England with evidence suggesting in vivo development of this unusual phenotype during prolonged intensive care of a patient. Here, we describe the genomic and molecular characteristics of this isolate, analysed alongside two clonally related isolates recovered from the same hospital using comparative genomics and bacteriological experiments.

## Methods

### Isolate collection and phenotyping

The *K. pneumoniae* isolate KpMVS1 was recovered from a lung biopsy specimen of an inpatient (hereafter, Patient 1) admitted to an intensive care unit (ICU) in England in 2021. The second *K. pneumoniae* isolate, KpMVR1, was recovered from a groin wound of the same patient 42 days later. During this ICU stay, the patient received a broad range of antimicrobials, including meropenem-vaborbactam, ciprofloxacin, and gentamicin; however, ceftazidime-avibactam and imipenem-relebactam were not used.

Species identification and carbapenemase-gene screening were performed using matrix-assisted laser desorption/ionisation time-of-flight (MALDI-TOF) mass spectrometry and the GeneXpert polymerase chain reaction (PCR) system (Cepheid, USA), respectively. Initial antimicrobial susceptibility testing (AST) was conducted by the hospital and interpreted according to European Committee on Antimicrobial Susceptibility Testing (EUCAST) guidelines. Both isolates were referred to the UKHSA Antimicrobial Resistance and Healthcare Associated Infections (AMRHAI) Reference Unit for variable number tandem repeat (VNTR) typing [31] and investigation of unusual antimicrobial resistance (AMR). Additionally, a KPC-*Kp* isolate KpMVS2—recovered from a rectal swab of another patient (Patient 2) in the same hospital in 2020 during an outbreak investigation and sharing the same VNTR profile as KpMVS1 and KpMVR1—was retrieved from AMRHAI’s culture collection for comparison. All three isolates underwent whole-genome sequencing (WGS) and AST, including determination of minimum inhibitory concentrations (MICs) for 20 antimicrobials (Table 1) and inhibition zone diameters for cefiderocol, interpreted according to EUCAST clinical breakpoints v15.0 [32].

**Table 1:** Antimicrobial minimum inhibitory concentrations (mg/L) determined by UKHSA’s AMRHAI Reference Unit and susceptibility interpretations (according to EUCAST clinical breakpoints v15.0 where applicable) of three *K. pneumoniae* clinical isolates. Abbreviations: MEM, meropenem; VAB, vaborbactam; IPM, imipenem; ETP, ertapenem; CET, ceftolozane; TZB, tazobactam; CFD, cefiderocol; FEP, cefepime; CAZ, ceftazidime; AVI, avibactam; CTX, cefotaxime; FOX, cefoxitin; TMC, temocillin; AMP, ampicillin; AMX, amoxicillin; CAV, clavulanate; PIP, piperacillin; ATM, aztreonam; AMK, amikacin; GEN, gentamicin; CST, colistin; CIP, ciprofloxacin; TIG, tigecycline; MB, monobactam. Susceptibility interpretations: R, resistant; I, susceptible, increased exposure; S, susceptible

	Carbapenem				Cephalosporin							Penicillin				MB	Aminoglycoside		Others		
Isolate	MEM-VAB	MEM	IPM	ETP	CET-TZB	CFD^*^	FEP	CAZ	CAZ-AVI	CTX	FOX^‡^	TMC^‡^	AMP	AMX-CAV	PIP-TZB	ATM	AMK^‡^	GEN^‡^	CIP	CST^‡^	TIG^‡^
KpMVR1	>256 (R)	>16 (R)	>128 (R)	>4 (R)	16 (R)	(S)	4 (I)	256 (R)	16 (R)	8 (R)	>64	>128	>32 (R)	>32 (R)	>64 (R)	16 (R)	2	0.5	>4 (R)	≤0.5	0.25
KpMVS1	0.064 (S)	>16 (R)	64 (R)	>4 (R)	>16 (R)	(S)	>32 (R)	128 (R)	1 (S)	64 (R)	>64	32	>32 (R)	>32 (R)	>64 (R)	>32 (R)	≤1	≤0.25	≤0.125 (S)	≤0.5	0.25
KpMVS2	0.032 (S)	16 (R)	16 (R)	>4 (R)	>16 (R)	(R)	>32 (R)	64 (R)	1 (S)	16 (R)	32	8	>32 (R)	>32 (R)	>64 (R)	>32 (R)	≤1	≤0.25	≤0.125 (S)	1	0.25

^*^ Susceptibility was determined using disc diffusion. ^‡^ Interpretations were not available according to EUCAST guidelines

### Whole-genome sequencing

Genomic DNA was extracted from overnight cultures using the GeneJET Kit (ThermoFisher Scientific, UK) according to the manufacturer’s protocol. Short-read sequencing was performed on a HiSeq 2500 system (Illumina, USA) in the UKHSA Colindale Sequencing Laboratory using a paired-end 101-bp protocol. Long-read sequencing was conducted on MinION R9.4.1 flow cells (Oxford Nanopore Technologies [ONT], UK), with libraries prepared using the ONT Rapid Barcoding Kit SQK-RBK004.

### Bioinformatics analysis

Illumina reads were trimmed and filtered with Trimmomatic v0.39 for a minimum per-read quality of Phred Q30 and minimum length of 50 bp [33]. Fast-mode basecalling and de-multiplexing of MinION reads was conducted by guppy v4 (ONT), followed by read trimming and filtering for a minimum per-read quality of Q10 and minimum length of 1 kbp with fastp v0.23.4 [34]. For species confirmation and contamination assessment, taxonomical profiling of processed Illumina and MinION reads were performed using Kraken v2.1.3, bracken v2.8, and a standard Kraken database built in September 2023 [35, 36].

Genomes of KpMVR1 and KpMVS2, with estimated MinION read depths of 185× and 243×, respectively, were assembled using hybracter v0.5.0 (Additional File 1) [37, 38]. For KpMVS1, which had an estimated MinION read depth of 64×, chromosomal and plasmid sequences were assembled using Raven v1.8.3 and plassembler, respectively, and were subsequently polished with MinION reads followed by Illumina reads, as for KpMVR1 and KpMVS2. Genome assemblies were assessed for quality using CheckM2 v1.0.2 and its database Uniref100/KO [39]. The average fold-coverage of each contig was estimated from Illumina and MinION reads, respectively, using mosdepth v0.3.9 [40].

The genome assemblies were annotated using bakta v1.9.2 and its standard database v5.1 [41]. Multi-locus sequence typing (MLST), serotype prediction, and virulence-factor detection were performed using Kleborate v3.1.3, which incorporated Kaptive v3.1.0 [42, 43]. Genome assemblies of all three isolates were submitted to the *Klebsiella* PasteurMLST database (bigsdb.pasteur.fr) for assignment of a new sequence type (ST) number. AMR genes were detected using AMRFinderPlus v3.12.8 with a minimum query coverage of 80% [44]. Clustered regularly interspaced short palindromic repeats (CRISPR) and CRISPR-associated (Cas) genes were predicted for chromosomes using CRISPRCasFinder [45]. For plasmids, replicon types were determined at a minimum of 80% nucleotide identity and coverage using PlasmidFinder v2.1 [46], and the mobility was predicted using mob_typer of MOB-suite v3.1.8 [47]. The copy number of each KPC-encoding plasmid was estimated by dividing the plasmid’s fold-coverage by that of its host’s chromosome. Transposons and insertion sequences (ISs) were identified using TnCentral Blast (blastn) and ISfinder, respectively [48, 49].

Chromosome and plasmids of KpMVR1 and KpMVS2 were compared against those of KpMVS1 using minimap v2.26 [50]. Identified genetic variants were annotated using snpEff v5.2 [51]. Gene Ontology terms were predicted from amino acid sequences using InterProScan v5.69–101.0 with sequence alignments filtered for ≥60% query coverages [52]. Impacts of point mutations on protein stability were predicted from wild-type protein structures in the UniProt database using Missense3D and DDMut [53–55]. The three-dimensional structure of the plasmid-encoded donor OM protein TraN was compared between KPC-encoding, IncFII-carrying plasmids pKpMVS1_1, pKpMVR1_1, and pKpMVS2_1 to estimate the impact of TraN alterations on the conjugation specificity and efficiency (Additional File 1) [38, 56]. Comparison and annotations of these three plasmids were visualised using BRIG v0.95 and Proksee [57, 58]. Gene synteny was illustrated using R package gggenes [59]. Genetic alterations in both KpMVR1 and KpMVS2 were considered unlikely to confer the unique AMR profiles of KpMVR1 and were therefore excluded from further investigation.

### Functional assessment

To determine and compare the impacts of *bla*_KPC-2_ and *bla*_KPC-157_ on β-lactam susceptibility in *K. pneumoniae*, KpMVS1’s KPC-2-encoding plasmid pKpMVS1_1 was introduced into the plasmid-free *K. pneumoniae* laboratory strain ICC8001 (MICs: meropenem, ≤0.06 mg/L; imipenem, 0.25 mg/L; aztreonam, ≤0.125 mg/L; ceftazidime and ceftazidime-avibactam, 0.25 mg/L) through conjugation, resulting in a transconjugant ICC8001_KPC-2_ [60]. Transgenic isolates ICC8001_KPC-157_ and KpMVS1_KPC-157_ were derived from ICC8001_KPC-2_ and KpMVS1, respectively, by introducing a 392A > G mutation (amino acid substitution: N131S) into *bla*_KPC-2_ through site-directed mutagenesis using primers KPC_invR (CAACGATATTCCTTGTTTG) and KPC_invF (GGCTCTGAAAATCATCTATTG). Moreover, isolates ICC8001_KPC-2/Δ*ompK36*_ and ICC8001_KPC-157/Δ*ompK36*_ were derived from ICC8001_KPC-2_ and ICC8001_KPC-157_, respectively, through seamless, markerless homologous recombination using mutagenesis vectors and a lambda-red based recombination system generated in previous work [60].

To predict the presence or absence of OmpK36 in KpMVR1’s OM, Sec-dependent signal peptides and their cleavage sites in translated *ompK36* alleles were compared between KpMVS1 and KpMVR1 using SignalP v6.0 [61]. To validate this prediction, OM proteins were purified from overnight cultures of KpMVS1, KpMVR1, KpMVS2, ICC8001, and an *ompK36*-knockout derivative, ICC8001_Δ*ompK36*_, separated by sodium dodecyl-sulfate polyacrylamide gel electrophoresis (SDS-PAGE), and visualised by Coomassie staining (Additional File 1) [38].

Both progenitor isolates (KpMVS1 and KpMVR1) and five transgenic derivatives (KpMVS1_KPC-157_, ICC8001_KPC-2_, ICC8001_KPC-157_, ICC8001_KPC-2/Δ*ompK36*_, and ICC8001_KPC-157/Δ*ompK36*_) were tested for susceptibility to meropenem, meropenem-vaborbactam, imipenem, imipenem-relebactam, imipenem-avibactam, ceftazidime, ceftazidime-avibactam, aztreonam, aztreonam-avibactam, and cefiderocol (in iron-depleted medium) using broth microdilution according to EUCAST guidelines [62, 63]. Any MIC change exceeding a two-fold difference between two isolates was considered notable.

## Results

### Phenotypes of isolates

Isolates KpMVS1 and KpMVR1, obtained 42 days apart from Patient 1 with recurrent *K. pneumoniae* infections, and KpMVS2, obtained from Patient 2 in the same hospital, were identified as *K. pneumoniae* by both MALDI-TOF and the WGS. In the ICU where KpMVS1 and KpMVR1 were recovered, all patients were screened for carriage of carbapenemase-producing Enterobacterales (CPE) on admission using PCR. The resistance profile of KpMVR1 was unique among all CPE isolates identified in the ICU during Patient 1’s stay.

Based on AST results from the AMRHAI Reference Unit (Table 1), KpMVR1 was resistant to meropenem-vaborbactam (MIC > 256 mg/L), ceftazidime-avibactam (MIC = 16 mg/L), and ciprofloxacin (MIC > 4 mg/L), whereas KpMVS1 and KpMVS2 were susceptible to these antimicrobials (MICs: meropenem-vaborbactam ≤ 0.064 mg/L; ceftazidime-avibactam 1 mg/L; ciprofloxacin ≤ 0.125 mg/L). KpMVS2 was resistant to cefiderocol (inhibition zone diameter: 19 mm) as determined using disc diffusion. Further AST revealed that the imipenem-relebactam MIC of KpMVR1 (512 mg/L, resistant) was 2048-fold higher than that of KpMVS1 (0.25 mg/L, susceptible) (Table 2). Moreover, KpMVR1 exhibited a > 4-fold increase in the temocillin MIC (>128 mg/L) and a > 8-fold reduction in the cefepime MIC (4 mg/L, susceptible, increased exposure) compared with KpMVS1 (temocillin: 32 mg/L; cefepime: >32 mg/L, resistant).

**Table 2:** Minimum inhibitory concentrations of β-lactam antimicrobials and susceptibility interpretations (according to EUCAST clinical breakpoints v15.0 where applicable) of progenitor and transgenic *K. pneumoniae* isolates determined in the experiments for functional assessment. Subscripts in isolate names indicate transgenic isolates and corresponding genotypes. Abbreviations: MEM, meropenem; VAB, vaborbactam; IPM, imipenem; REL, relebactam; ATM, aztreonam; AVI, avibactam; CAZ, ceftazidime; CFD, cefiderocol, tested in iron-depleted Muelle-Hinton broth; NT, not tested. Interpretations of antimicrobial susceptibility: R, resistant; I, susceptible, increased exposure; S, susceptible. Notations: *ompK36*fs, frameshifted *ompK36*; Δ*ompK36*, deletion of *ompK36*

Isolate	Genotype		Minimum Inhibitory Concentration (mg/L) and interpretation									
			MEM	MEM-VAB	IPM	IPM-REL	IPM-AVI	ATM	ATM-AVI	CAZ	CAZ-AVI	CFD
KpMVR1	*bla*_KPC-157_	*ompK36*fs	512 (R)	256 (R)	512 (R)	512 (R)	NT	16 (R)	4 (S)	8 (R)	8 (S)	0.5 (S)
KpMVS1	*bla*_KPC-2_	*ompK36*	32 (R)	≤0.06 (S)	32 (R)	0.25 (S)	≤0.5	512 (R)	0.25 (S)	32 (R)	0.5 (S)	0.25 (S)
KpMVS1_KPC-157_	*bla*_KPC-157_	*ompK36*	16 (R)	≤0.06 (S)	8 (R)	8 (R)	≤0.5	2 (I)	0.25 (S)	1 (S)	0.25 (S)	≤0.06 (S)
ICC8001_KPC-2_	*bla*_KPC-2_	*ompK36*	16 (R)	≤0.06 (S)	16 (R)	0.25 (S)	≤0.5	512 (R)	0.125 (S)	32 (R)	0.25 (S)	0.25 (S)
ICC8001_KPC-157_	*bla*_KPC-157_	*ompK36*	16 (R)	≤0.06 (S)	4 (I)	4 (R)	≤0.5	1 (S)	0.125 (S)	0.5 (S)	0.125 (S)	≤0.06 (S)
ICC8001_KPC-2/Δ*ompK36*_	*bla*_KPC-2_	Δ*ompK36*	256 (R)	2 (S)	256 (R)	2 (S)	NT	>1024 (R)	0.25 (S)	16 (R)	0.5 (S)	0.25 (S)
ICC8001_KPC-157/Δ*ompK36*_	*bla*_KPC-157_	Δ*ompK36*	256 (R)	4 (S)	256 (R)	128 (R)	NT	4 (I)	0.25 (S)	1 (S)	0.5 (S)	0.25 (S)

### Genetic characteristics of isolates

All three isolates belonged to *K. pneumoniae* clone ST8134, a single-locus variant of ST240, and were predicted to share the O1αβ,2β O-antigen type and K62 capsular polysaccharide type. Sequence lengths, plasmid replicons, and AMR genes determined in hybrid genome assemblies are summarised in Table 3. KpMVR1 and KpMVS1 shared the same plasmid types IncFII/repB(R1701), IncFII(pMET), Col(pHAD28), and Col(pHAD28)/Col440II, whereas KpMVS2 possessed unique plasmid types IncFII/IncR and IncFII(pKP91)/FIB(K).

**Table 3:** Genetic characteristics of three *K. pneumoniae* clinical isolates. The plasmid type refers to the haplotype of plasmid replicons. Each hit of plasmid replicons in this table covered the full length of its reference sequence in the PlasmidFinder database

Isolate	Sequence	Category	Length (bp)	Plasmid type	Nucleotide identity to template	Plasmid mobility	AMR gene
KpMVS1	KpMVS1	Chromosome	5,404,514				*bla*_SHV-36_, *fosA10*
	pKpMVS1_1	Plasmid	111,397	IncFII/repB(R1701)	IncFII(pKP91): 100%; repB(R1701): 99.52%	Conjugative	*bla*_KPC-2_
	pKpMVS1_2	Plasmid	41,868	IncFII(pMET)	IncFII(pMET): 98.09%	Non-mobilisable	
	pKpMVS1_3	Plasmid	4,809	Col(pHAD28)	Col(pHAD28): 92.37%	Non-mobilisable	
	pKpMVS1_4	Plasmid	4,439	Col(pHAD28)/Col440II	Col(pHAD28): 93.13%; Col440II: 97.52%	Mobilisable	
	pKpMVS1_5	Plasmid	3,258	Unknown	Not detected	Non-mobilisable	
	pKpMVS1_6	Plasmid	1,917	Col(pHAD28)	Col(pHAD28): 100%	Mobilisable	
KpMVR1	KpMVR1	Chromosome	5,292,801				*bla*_SHV-36_, *fosA10*
	pKpMVR1_1	Plasmid	111,174	IncFII/repB(R1701)	IncFII(pKP91): 100%, repB(R1701): 99.52%	Conjugative	*bla*_KPC-157_
	pKpMVR1_2	Plasmid	41,868	IncFII(pMET)	IncFII(pMET): 98.09%	Non-mobilisable	
	pKpMVR1_3	Plasmid	4,809	Col(pHAD28)	Col(pHAD28): 92.37%	Non-mobilisable	
	pKpMVR1_4	Plasmid	4,439	Col(pHAD28)/Col440II	Col(pHAD28): 93.13%; Col440II: 97.52%	Mobilisable	
	pKpMVR1_5	Plasmid	3,258	Unknown	Not detected	Non-mobilisable	
	pKpMVR1_6	Plasmid	1,917	Col(pHAD28)	Col (pHAD28): 100%	Mobilisable	
KpMVS2	KpMVS2	Chromosome	5,354,507				*bla*_SHV-36_, *fosA10*
	pKpMVS2_1	Plasmid	116,795	IncFII/IncR	IncFII(pKP91): 100%; IncR: 99.6%	Conjugative	*bla*_KPC-2_
	pKpMVS2_2	Plasmid	41,868	IncFII(pMET)	IncFII(pMET): 98.09%	Non-mobilisable	
	pKpMVS2_3	Plasmid	4,808	Col(pHAD28)	Col (pHAD28): 92.37%^*^	Non-mobilisable	
	pKpMVS2_4	Plasmid	4,187	Col(pHAD28)	Col (pHAD28): 92.37%^*^	Non-mobilisable	
	pKpMVS2_5	Plasmid	3,258	Unknown	Not detected	Non-mobilisable	
	pKpMVS2_6	Plasmid	1,917	Col(pHAD28)	Col (pHAD28): 100%	Mobilisable	
	pKpMVS2_7	Plasmid	240,297	IncFII(pKP91)/IncFIB(K)	IncFII(pKP91): 84.98%; IncFIB(K): 98.93%	Conjugative	

^*^ Two hits of the Col(pHAD28) template sequence in the PlasmidFinder database differed between pKpMVS2_3 and pKpMVS2_4 by seven nucleotide substitutions (95% nucleotide identity) despite their same percent identity to the template

Abbreviation: AMR, antimicrobial resistance

KpMVS1 carried *bla*_KPC-2_ on the 111.4-kbp IncFII(pKP91)/repB(R1701) plasmid pKpMVS1_1. A plasmid of the same type was identified in KpMVR1 (pKpMVR1_1, 111.2 kbp) and carried *bla*_KPC-157_, which differed from *bla*_KPC-2_ by a single missense mutation (392A > G) resulting in an N131S amino acid substitution within the enzyme’s active site (NCBI Protein accessions: KPC-2, WP_004199234.1; KPC-157, WP_259115967.1) [64], where the N131 residue binds to relebactam, avibactam, and vaborbactam through a hydrogen bond [65–67]. Notably, pKpMVR1_1 differed from pKpMVS1_1 by 285 nucleotide substitutions, 14 deletions, and three insertions. These variants were concentrated in two genomic regions involved in plasmid transfer and maintenance (Supplementary Fig. 1, Additional File 2) [68], suggesting recombination between plasmids. Another plasmid type, IncFII(pKP91)/IncR, in KpMVS2 carried *bla*_KPC-2_. All these KPC-encoding plasmids were predicted to be conjugative (relaxase type: MOBF; mating pair formation type: MPF_F), and each carried an intact variant of the Tn*4401a* transposon harbouring *bla*_KPC-2_ or *bla*_KPC-157_, with an ATTGA target site duplication and 1–2 single-nucleotide polymorphisms (SNPs) between each pair of Tn*4401a* variants (Supplementary Fig. 1, Additional File 2; Supplementary Tables 1 and 2, Additional File 3) [38, 69–71]. The comparison between fold-coverages of contigs suggested that each of these three isolates carried a single copy of the KPC-encoding plasmid. Other AMR genes detected were chromosomal β-lactamase gene *bla*_SHV_ (variant *bla*_SHV-36_) and fosfomycin resistance gene *fosA10*, which are both intrinsic to *K. pneumoniae* [72–74].

**Fig. 1:**
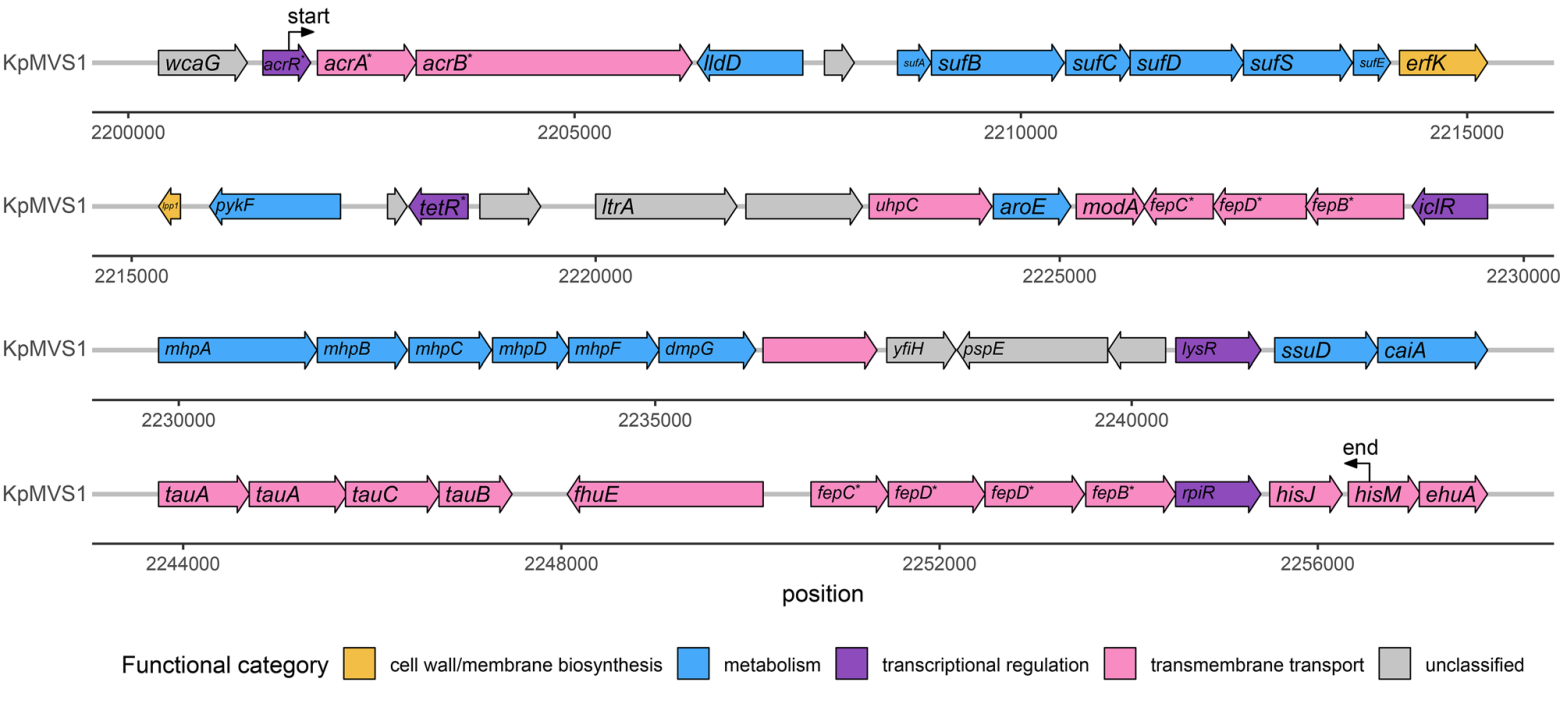
Genetic structure of a 54.7-kbp region in KpMVS1 that was deleted in KpMVR1 (Table 4). Labels “start” and “end” indicate boundaries of the deleted region. Genes without known names are not labelled. Each asterisk indicates an allele from a named gene family

The chromosome of KpMVR1 differed from that of KpMVS1 by six SNPs, seven insertions, four small deletions (1–15 bp), and four large deletions (Fig. 1; Supplementary Figs. 2,3,4,5, Additional File 2; Supplementary Tables 3,4,5,6, Additional File 3) [68, 71]. Seven variants were also identified in KpMVS2 (Table 4), which differed from KpMVS1 by 129 SNPs, 13 insertions, and seven deletions. The 19.7-kbp and 4.9-kbp deletions in KpMVR1 may have been mediated by IS elements, as previously reported in *Escherichia coli* (Supplementary Figs. 3 and 5, Additional File 2) [68, 75]. Notably, KpMVR1 exhibited a 54.7-kbp deletion encompassing operons encoding an AcrAB-like efflux pump in the resistance-nodulation-division (RND) transporter family and an additional ABC-type Fe^3+^-siderophore transport system present in both KpMVS1 and KpMVS2 (Fig. 1; Supplementary Table 3, Additional File 3) [71]. All three isolates carried a single copy of the *acrAB* operon and *tolC* gene, which together produce the AcrAB-TolC multidrug efflux pump. However, the permease AcrB in KpMVR1 harboured a destabilising L667R mutation located outside the protein’s transmembrane domains.

**Table 4:** Chromosomal genetic variation in isolate KpMVR1 identified via comparison against its progenitor KpMVS1. Coordinates refer to locations in the reference sequence of the KpMVS1 chromosome. Variants shared by both KpMVR1 and KpMVS2 against their common reference sequence of KpMVS1 are indicated by asterisks following the coordinates. The “^” sign indicates an insertion between two consecutive bases in the reference sequence

Location	Locus	Product	Variant type	DNA change	Protein change
455179	*rrl*	23S rRNA	Substitution	G > T	
1050491 – 1070213	Multiple	Type I-E CRISPR-Cas system, *etc*. (Supp. Fig. 3, Supp. Table 4)	Deletion	Deletion of 19,723 bp	Loss of production
1113441 – 1113446	*flhA*	Formate hydrogenlyase transcriptional activator	Deletion	Deletion of 6 bp	L367Del, T368Del
1218110^1218111*	*rrl*	23S rRNA	Insertion	Insertion of a base G	
1578603	*gyrA*	DNA topoisomerase (ATP-hydrolyzing) subunit A	Substitution	248C > A	S83Y
1588108^1588109	*ompK36*	Outer membrane porin OmpK36	Insertion	Insertion of IS*Ec68*	Amino acid substitutions
1724677^1724678*	*xylB*	Xylulose kinase	Insertion	Insertion of a base G	I236fs
1792900	*rfbD*	UDP-galactopyranose mutase	Substitution	578T > A	M193K
1819375^1819376	Intergenic		Insertion	Insertion of a base C	
2183835^2183836*	Intergenic		Insertion	Insertion of a base T	
2183837*	Intergenic		Substitution	A > T	
2201799 – 2256547	Multiple	Multiple products including transporters (Fig. 1, Supp. Table 3)	Deletion	Deletion of 54,749 bp	Loss of production
2759671 – 2759685	*marR*	Multiple-AMR (Mar) transcriptional repressor MarR	Deletion	Deletion of bases 263–277	P88–D92Del, K93Q
3157221^3157222*	Intergenic		Insertion	Insertion of a base C	
3189754 – 3223285*	Multiple	Multiple products (Supp. Fig. 4, Supp. Table 5)	Deletion	Deletion of 33,532 bp	Loss of production
3274833	*phoQ*	Two-component system sensor histidine kinase	Substitution	C > T	T156I
3580334 – 3585227*	Multiple	IS*3 H* composite transposon (Supp. Fig. 5, Supp. Table 6)	Deletion	Deletion of 4,894 bp	Loss of production
4104884	*acrB*	Multidrug efflux RND transporter permease subunit AcrB	Substitution	*T* > G	L667R
4262704^4262705	*ecpR*	Regulator protein EcpR	Insertion	Insertion (2 bp)	I115fs
4723928	*rrl*	23S ribosomal RNA	Deletion	Deletion of the base C	
5349457	*rrl*	23S ribosomal RNA	Deletion	Deletion of the base C	

Abbreviations: CRISPR, clustered regularly interspaced short palindromic repeats; Cas: CRISPR-associated genes; Del, deletion; Ins, insertion; fs, frameshift

As for the biosynthesis of siderophores and transport of the iron-siderophore complex—which together facilitate cefiderocol to penetrate the OM [76]—KpMVS1, KpMVR1, and KpMVS2 were predicted to possess complete enterobactin production and iron-enterobactin transport systems. None of the yersiniabactin, colibactin, aerobactin, or salmochelin loci were detected, producing a Kleborate virulence score of zero [42]. All three isolates shared the same 19-kbp chromosomal region harbouring a cluster of enterobactin-synthesising genes *entA–F and entH*, enterobactin-exporter gene *entS*, and iron-enterobactin transporter genes *fepA–D* and *fepG*.

Notably, an intact, iron-transporting *fec* operon [77] was identified on the 240-kbp IncFII (pKP91)/IncFIB(K) plasmid pKpMVS2_7 (bases 85,801–93,352 in GenBank record CP182593.1), which is unique to KpMVS2 (Table 3). This plasmid also encoded resistance to silver, copper, nickel, and heat. The plasmid-borne *fec* operon may therefore account for KpMVS2’s unique cefiderocol resistance compared with KpMVR1 and KpMVS1 (Table 1) [78]. No plasmid homologous to pKpMVS2_7 was identified in the NCBI nucleotide database using megaBLAST, whereas the complete *fec* operon was detected in 945 *K. pneumoniae* plasmids at >99.6% nucleotide identity on 16 February 2026, suggesting horizontal transfer of this operon between plasmids.

Compared with the ciprofloxacin-susceptible isolates KpMVS1 and KpMVS2, KpMVR1 harboured a nucleotide substitution 248C > A in the DNA gyrase gene *gyrA*, generating the GyrA mutation S83Y, which is known to reduce ciprofloxacin susceptibility [79]. The three isolates also carried a single copy of the *marRAB* operon. However, KpMVR1 exhibited a unique 15-bp in-frame deletion in the non-essential transcriptional repressor gene *marR* within the *marRAB* operon, causing a loss of five amino acids and an amino acid substitution within the DNA-binding region of MarR (Table 4) [80].

**Fig. 2:**
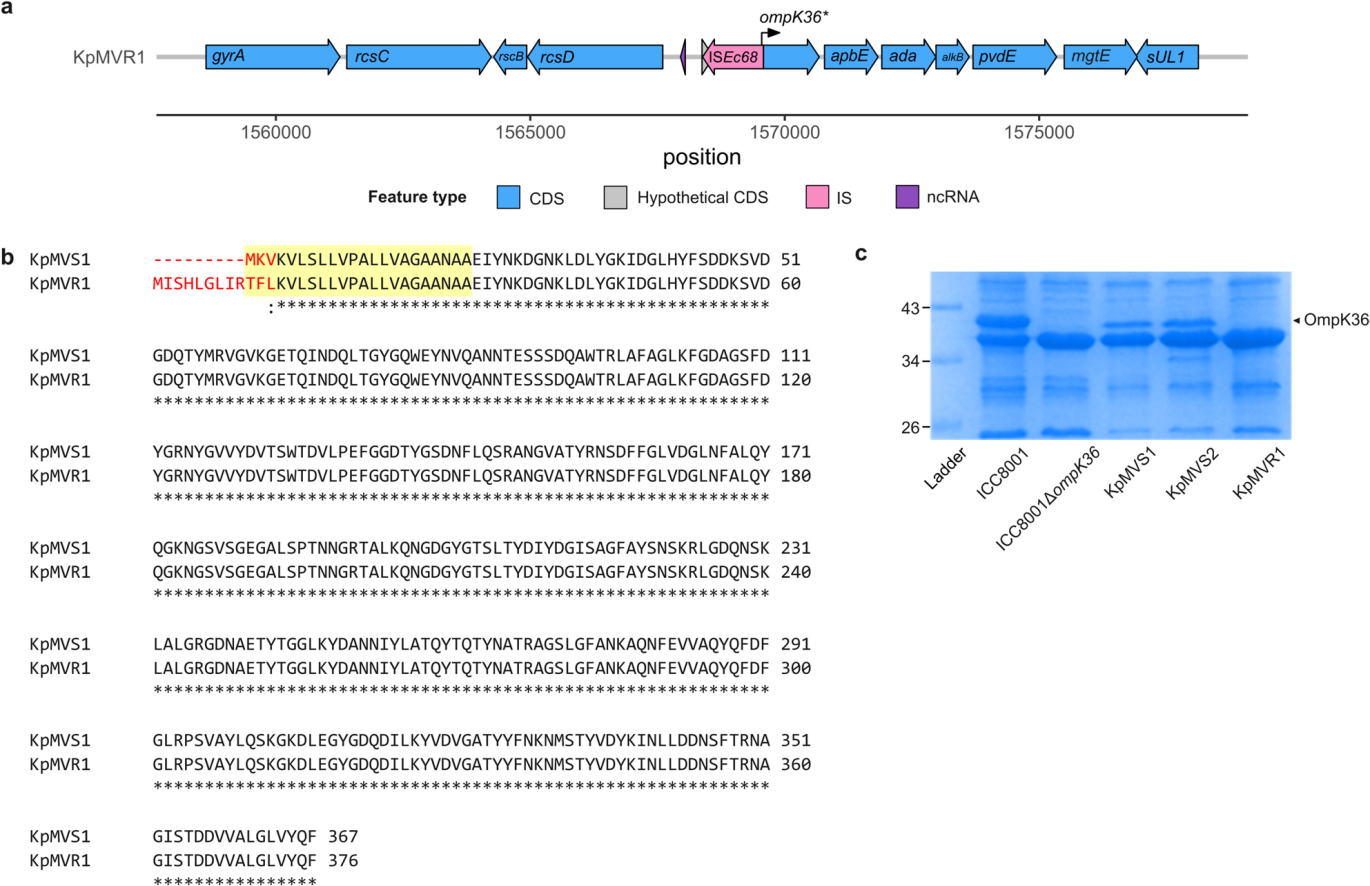
IS*Ec68*-mediated disruption of *ompK36* in isolate KpMVR1. (**a**) Genetic environment of the disrupted *ompK36*. The arrow labelled “ompK36*” denotes the upstream-shifted open reading frame caused by the insertion of IS*Ec68*. Abbreviations: CDS, coding sequence; IS, insertion sequence; ncRNA, non-coding RNA. (**b**) Comparison of predicted OmpK36 sequences using Clustal Omega (www.ebi.ac.uk/jdispatcher/msa/clustalo). Mismatches are highlighted in red, and the 22 N-terminal amino acids signal sequence of OmpK36 are indicated by the yellow shade. (**c**) Coomassie-stained polyacrylamide gel electrophoresis of outer membrane proteins to confirm the absence of OmpK36 in KpMVR1 and the *ompK36*-knockout isolate ICC8001_Δ*ompK36*_. See Additional File 4 for the original gel image

Both the KpMVS1 and KpMVS2 genomes harboured three identical copies of the IS*5*-family insertion sequence IS*Ec68*, whereas the KpMVR1 genome harboured four. Notably, seven bases at the 5’ end of *ompK36* in KpMVR1 were truncated by this additional copy of IS*Ec68*, producing a frameshift mutation that replaced the first three amino acids at the N-terminal of OmpK36 with 12 amino acids (Fig. 2). The native N-terminal 21 amino acids of OmpK36 encode a Sec-dependent signal sequence (UniProtKB accession: A0A0H3H0Y2), which is essential for translocation of OmpK36 to the inner membrane of *K. pneumoniae* (Fig. 2b) [81]. The signal sequence is subsequently cleaved and OmpK36 is then folded and inserted into the OM (where the protein is functionally active as a porin) in a Bam-complex dependent fashion, a process facilitated by a C-terminal recognition sequence [82]. Whilst *ompK36* from KpMVS1 and KpMVS2 is predicted to encode a complete Sec-dependent signal sequence, the 12 amino acids insertion combined with the deletion of three amino acids in OmpK36 from KpMVR1 is predicted to hinder this protein’s translocation to the OM according to the disrupted signal sequence (Supplementary Fig. 6, Additional File 2) [68]. These predictions were confirmed by the SDS-PAGE, which showed a Coomassie-stained band corresponding to OmpK36 in KpMVS1 but not in KpMVR1 (Fig. 2c and Additional File 4) [83], indicating that disruption of the Sec-dependent signal sequence of OmpK36 is functionally equivalent to deletion of *ompK36*.

Regarding plasmid-encoded TraN proteins, TraN_pKpMVR1_1_ and TraN_pKpMVS2_1_ were identical (NCBI protein accession: WP_049192820.1) and differed from TraN_pKpMVS1_1_ (WP_436914186.1) by six amino acid substitutions (Supplementary Table 7, Additional File 3) [71]. Phylogenetic analysis revealed that these proteins belonged to the specialist TraNβ group (Supplementary Fig. 7, Additional File 2) [68], which has a narrow host range [56, 84]. Pairwise structural comparison between TraN_pKpMVR1_1_ (TraN_pKpMVS2_1_), TraN_pKpMVS1_1_, and the prototype TraNβ protein TraN_pKpQIL_ showed high consistency (Supplementary Fig. 8, Additional File 2) [68], and no amino acid substitution occurred in the characteristic distal β-hairpin (Supplementary Fig. 9, Additional File 2) [68], suggesting that the variation in TraN sequences across plasmids pKpMVR1_1, pKpMVS1_1, pKpMVS2_1, and pKpQIL is unlikely to affect the conjugation specificity [85].

### Impact of genetic alterations on antimicrobial resistance

The substitution of *bla*_KPC-2_ with *bla*_KPC-157_ in KpMVS1 (producing KpMVS1_KPC-157_) and the transconjugant ICC8001_KPC-2_ (producing ICC8001_KPC-157_) did not affect the susceptibility to meropenem, meropenem-vaborbactam, or imipenem-avibactam but led to a fourfold reduction in the imipenem MIC and a 16- to 32-fold increase in the imipenem-relebactam MIC (Table 2). Moreover, this allelic substitution resulted in a 256- to 512-fold reduction in the aztreonam MIC, a 32- to 64-fold reduction in the ceftazidime MIC, and a ≥4-fold reduction in the cefiderocol MIC but had no effect on the MICs of aztreonam-avibactam or ceftazidime-avibactam. Notably, for KpMVS1_KPC-157_, the imipenem MIC equaled the imipenem-relebactam MIC; the same was observed for ICC8001_KPC-157_ (Table 2). These findings suggest that KPC-157 has a weaker capacity to hydrolyse imipenem, aztreonam, ceftazidime, and cefiderocol than KPC-2, and that—unlike vaborbactam and avibactam, which inhibit both KPC variants—relebactam inhibits KPC-2 but not KPC-157, which is consistent with a previous report [65].

Knocking out *ompK36* from the ICC8001 chromosome (ICC8001_KPC-2/Δ*ompK36*_ and ICC8001_KPC-157/Δ*ompK36*_) resulted in a 16-fold increase in meropenem and imipenem MICs, a >33-fold increase in meropenem-vaborbactam MIC, an 8- to 32-fold increase in imipenem-relebactam MIC, and a more than twofold increase in aztreonam MIC (Table 2). These findings support the role of OmpK36 as an entry route for β-lactams and β-lactamase inhibitors across the OM [25]. However, when comparing MICs of ceftazidime, ceftazidime-avibactam, and cefiderocol before and after the *ompK36* knockout in ICC8001_KPC-2_ and ICC8001_KPC-157_, only two pairs exhibited notable increases (from 0.125 mg/L to 0.5 mg/L for ceftazidime-avibactam, and from ≤0.06 mg/L to 0.25 mg/L for cefiderocol), while others remained unchanged, suggesting alternative routes for avibactam’s entry. More broadly, comparisons of β-lactam MICs with and without β-lactamase inhibitors for KpMVR1, ICC8001_KPC-2/Δ*ompK36*_, and ICC8001_KPC-157/Δ*ompK36*_ (Table 2) indicate that these inhibitors penetrated the OM via routes other than OmpK36, effectively inhibiting β-lactamases.

The chromosomes of KpMVR1, KpMVS1, and ICC8001 derivatives harboured the same cluster of *ent* and *fep* genes within a 19-kbp region encoding an ABC-type Fe^3+^-siderophore transporter associated with cefiderocol susceptibility [76]. These isolates did not exhibit any notable difference in cefiderocol MICs despite KpMVR1’s loss of the 54.7-kbp chromosomal region harbouring *fep*-like genes (Supplementary Table 3, Additional File 3) [71], suggesting alternative entry routes of cefiderocol.

## Discussion

In the UK, KPC-*Kp* clinical isolates resistant to meropenem-vaborbactam, imipenem-relebactam, and ceftazidime-avibactam had not been documented prior to this study, despite UKHSA guidance to submit metallo-carbapenemase-negative isolates exhibiting resistance to any of these agents to the AMRHAI Reference Unit for investigation. The identification of KpMVR1 in a seriously ill patient therefore raised concern about the potential emergence and transmission of resistance to these three agents, which are reserved for highly selected patients [86].

The small number of chromosomal SNPs (*n* = 6) and indels (*n* = 10; ≤15 bp each) identified in KpMVR1 when compared with KpMVS1, together with Patient 1’s exposure to meropenem, meropenem-vaborbactam, and fluoroquinolones, and the unique antibiogram of KpMVR1 in the ICU suggest in vivo development of resistance to meropenem-vaborbactam, ceftazidime-avibactam, imipenem-relebactam, and ciprofloxacin in the same bacterial strain during the patient’s prolonged hospital stay. Similar within-host evolution of resistance to imipenem-relebactam, meropenem-vaborbactam, or ceftazidime-avibactam in KPC-*Kp* have been reported during extended hospitalisation involving complex antimicrobial regimens, including combinations such as ceftazidime-avibactam plus meropenem or meropenem-vaborbactam [21, 87].

This study also suggests within-patient emergence of *bla*_KPC-157_ through a spontaneous point mutation in *bla*_KPC-2_ during prolonged intensive care with exposure to a broad range of antimicrobials. To date, *bla*_KPC-157_ has been sporadically reported in Enterobacterales and is associated with distinct plasmids. Two *bla*_KPC-157_-positive *K. pneumoniae* ST11 clinical isolates, resistant to imipenem-relebactam and ceftazidime-avibactam, were recovered from two patients in China between 2023 and 2024 [88]. In both isolates, *bla*_KPC-157_ was carried by the same IncFII(pSDP9R) plasmid (GenBank accessions: CP148070.1 and CP152056.1), as we determined using PlasmidFinder v2.1. Furthermore, *bla*_KPC-157_ has also been identified in an IncFII(Yp)-like plasmid of a *K. huaxiensis* isolate from hospital wastewater in China in 2022, and in an IncN2 plasmid of a *Citrobacter freundii* isolate from a sewage treatment plant in Brazil [88, 89]. Given the consistent association between *bla*_KPC-157_ and Tn*4401* isoforms in all aforementioned cases, continuous surveillance of KPC variants across clinical settings and environmental reservoirs is warranted to monitor emerging resistance mechanisms.

KPC-2 confers carbapenem resistance in Gram-negative bacteria but can be effectively inhibited by vaborbactam, avibactam, and relebactam [90, 91]. We have experimentally determined the effect of carbapenemase KPC-157 on the susceptibility to carbapenems and cephalosporins, with or without β-lactamase inhibitors. Our results indicate that KPC-157 behaves similarly to KPC-2 in interactions with meropenem and meropenem-vaborbactam. Therefore, the presence of *bla*_KPC-157_ in the single-copy plasmid pKpMVR1_1 alone cannot explain the high-level meropenem-vaborbactam resistance observed in KpMVR1. Notably, KPC-157 appears less capable of hydrolysing imipenem, aztreonam, ceftazidime, and cefiderocol than KPC-2, and is inhibited by vaborbactam and avibactam but not by relebactam.

Isolate KpMVR1 exhibited genetic changes potentially affecting the antimicrobial permeability of its OM compared with KpMVS1. The IS*Ec68*-induced frameshift mutation impedes OmpK36’s translocation to the OM, decreasing the influx of β-lactams and β-lactamase inhibitors into the periplasm and resulting in elevated MICs of carbapenems and cephalosporins, with and without β-lactamase inhibitors (Table 2). IS*Ec68* has been sporadically reported to interrupt *ompK36* and *mgrB* in *K. pneumoniae* clinical and wastewater isolates, reducing the susceptibility of host bacteria to β-lactams and colistin, respectively [92–94]. Our finding further supports IS*Ec68*’s association with AMR.

The *gyrA* S83Y mutation and loss of OmpK36 function in KpMVR1 are associated with ciprofloxacin resistance [79, 95]. Further studies are needed to assess the impact of the identified *marR* and *acrB* mutations on antimicrobial susceptibility of *K. pneumoniae* and to explore potential interactions between the MarR and AcrB variants. For example, *marR* inactivation is known to upregulate the AcrAB-TolC efflux pump, conferring low-level cross-resistance to antimicrobials including β-lactams and ciprofloxacin [96].

This study was limited to three clonally related *K. pneumoniae* isolates, of which only KpMVR1 exhibited elevated MICs of meropenem-vaborbactam, imipenem-relebactam, aztreonam-avibactam, and ceftazidime-avibactam. KpMVR1 carried multiple AMR-associated genetic alterations. Although the individual effects of *bla*_KPC-157_ and Δ*ompK36* on antimicrobial susceptibility were determined, the transgenic derivatives did not fully reproduce MIC increases observed in KpMVR1, suggesting involvement of additional genetic or regulatory mechanisms. Broader surveillance of *K. pneumoniae* with resistance profiles like KpMVR1 is warranted to elucidate underlying resistance mechanisms.

A review of national surveillance data showed low frequencies of ceftazidime-avibactam resistance in the UK between 2016 and 2020 [15]. However, timely identification and reporting of resistance to β-lactam/β-lactamase inhibitor combinations remain essential through UKHSA’s SGSS and referral of resistant isolates to the AMRHAI Reference Unit. We also emphasise screening Enterobacterales genomes for the mobile *fec* operon, which confers cefiderocol resistance and was not detected by AMRFinderPlus in our study. Furthermore, our findings underscore the importance of monitoring within-patient changes in antimicrobial susceptibility profiles of bacterial pathogens during antimicrobial therapy.

## Electronic supplementary material

Below is the link to the electronic supplementary material.

Supplementary Material 1: Additional File 1

Supplementary Material 2: Additional File 2

Supplementary Material 3: Additional File 3

Supplementary Material 4: Additional File 4

## Data Availability

Illumina and MinION sequencing reads, genome assemblies, and anonymised metadata of isolates KpMVS1 (BioSample accession: SAMN46778009), KpMVR1 (SAMN46778010), and KpMVS2 (SAMN46778011) are available in the National Center for Biotechnology Information [97] under BioProject PRJNA1084250 [98]. The assemblies are also accessible (website login required) in the *Klebsiella* PasteurMLST database [99] under ids 75608 (KpMVS1) [100], 75609 (KpMVR1) [101], and 75610 (KpMVS2) [102].

## Abbreviations

AMR: Antimicrobial resistance
AMRHAI: Antimicrobial Resistance and Healthcare Associated Infections
AST: Antimicrobial susceptibility testing
CPE: Carbapenemase-producing Enterobacterales
CRISPR: Clustered regularly interspaced short palindromic repeats
ESBL: Extended-spectrum β-lactamase
EUCAST: European Committee on Antimicrobial Susceptibility Testing
ICU: Intensive care unit
IS: Insertion sequence
KPC: *Klebsiella pneumoniae* carbapenemase
KPC-*Kp*: KPC-producing *K. pneumoniae*
MIC: Minimum inhibitory concentration
MLST: Multi-locus sequence typing
NCBI: National Center for Biotechnology Information
ONT: Oxford Nanopore Technologies
OM: Outer membrane
MALDI-TOF: Matrix-assisted laser desorption/ionisation time-of-flight
PCR: Polymerase chain reaction
RND: Resistance-nodulation-division
SDS-PAGE: Sodium dodecyl-sulfate polyacrylamide gel electrophoresis
SGSS: Second Generation Surveillance System
SNP: Single-nucleotide polymorphism
ST: Sequence type
UK: United Kingdom
UKHSA: United Kingdom Health Security Agency
VNTR: Variable number tandem repeat
WGS: Whole-genome sequencing
