# Supplementary material for: Genomic and molecular characterisation of a KPC-producing Klebsiella pneumoniae clinical isolate resistant to meropenem-vaborbactam, imipenem-relebactam, and ceftazidime-avibactam
Source: BMC Genom Data. 2026 May 9;27:37. doi: 10.1186/s12863-026-01421-x (PMC13182041; doi:10.1186/s12863-026-01421-x)
Supplement: Supplementary file 1 — Supplementary Material 1: Additional File 1 [file 12863_2026_1421_MOESM1_ESM.docx]

Supplementary methods

**Genomic and molecular characterisation of a KPC-producing *Klebsiella pneumoniae* clinical isolate resistant to meropenem-vaborbactam, imipenem-relebactam, and ceftazidime-avibactam**

Yu Wan, Joshua L. C. Wong, Julia Sanchez-Garrido, Wen Wen Low, Jane F. Turton, Fabio Morecchiato, Ilaria Baccani, Kirsty Dodgson, Gian Maria Rossolini, Neil Woodford, Gad Frankel, Elita Jauneikaite, Danièle Meunier, and Katie L. Hopkins

**February 2026**

# Modules of hybracter v0.5.0

Assemblers: Flye v2.9.3 and plassembler v1.5.0; sequence re-orientator: dnaapler v0.5.1; long-read polisher: medaka v1.8.0, short-read polishers: pypolca v0.2.1 and polypolish v0.5.0 [1–5].

# Comparative structural analysis of TraN

Amino acid sequences of TraN_pKpMVS1_1_, TraN_pKpMVR1_1_, and TraN_pKpMVS2_1_ were extracted from the sequence annotations of plasmids pKpMVS1_1 (locus tag: WAS92_RS00545), pKpMVR1_1 (ACNQKT_RS26595), and pKpMVS2_1 (ACNQKS_RS28350), respectively. To contextualise these three proteins, the previously described TraN variants TraN_pKpQI_ (NCBI protein accession: ARQ19727.1), TraN_MV2_ (BAS44060.1), TraN_R100-1_ (ABD60034.1), TraN_pSLT_ (AAL23498.1), TraN_F_ (WP_000821835.1), TraN_MV1_ (ANZ89826.1), TraN_MV3_ (WP_001398575.1) were downloaded from the NCBI Protein database ([www.ncbi.nlm.nih.gov/protein](http://www.ncbi.nlm.nih.gov/protein)) [6]. These 10 amino acid sequences were aligned with the ClustalW algorithm [7], and subsequently, a neighbour-joining phylogenetic tree was generated from the multi-sequence alignment, with the Poisson correction method as implemented in MEGA11 [8]. The phylogenetic tree was visualised using iTOL v7.2 [9].

Three-dimensional structures of TraN_pKpMVS1_1_, TraN_pKpMVR1_1_ (identical to TraN_pKpMVS2_1_), and TraN_pKpQI_ were predicted using AlphaFold 3 with its default parameters on AlphaFold Server (alphafoldserver.com) [10]. The top-ranked (model 0) structural models of TraN proteins were visualised using UCSF ChimeraX v1.9 [11]. Superimposition analysis of these models was performed with ChimeraX’s Matchmaker tool using default settings, including the use of the “best-aligning” or “bb” chain-pairing method, the Needleman-Wunsch alignment algorithm, and the BLOSUM-62 similarity matrix.

# Validation of predicted OmpK36 translocation

Outer membrane (OM) proteins were purified by resuspending overnight LB-Miller cultures (VWR, USA) of KpMVS1, KpMVR1, and KpMVS2 in 1M HEPES (pH 7.4) and sonicating at 25% amplitude for ten 10-second bursts with 15-second intervals (Model 705 Sonic Dismembrator, Fisher Scientific). Isolates ICC8001 and its *ompK36*-knockout derivative, ICC8001_Δ_*_ompK36_*, served as positive and negative controls, respectively. After separating cellular debris by centrifugation, OM proteins were obtained by centrifugation at 14,000×g for 30 minutes and resuspended in 2% sarcosine/HEPES for 30 minutes at room temperature. All steps were performed at 4°C on ice to preserve protein integrity unless otherwise specified. For visualisation, 10 μg of OM proteins per isolate was separated by SDS-PAGE using 12% acrylamide gels, stained with Coomassie solution (Sigma-Aldrich, USA), and imaged on a ChemiDoc XRS+ system (Bio-Rad, USA).

# References

1. Bouras G, Sheppard AE, Mallawaarachchi V, Vreugde S. Plassembler: an automated bacterial plasmid assembly tool. Bioinformatics. 2023;39:btad409. https://doi.org/10.1093/bioinformatics/btad409.

2. Bouras G, Grigson SR, Papudeshi B, Mallawaarachchi V, Roach MJ. Dnaapler: A tool to reorient circular microbial genomes. Journal of Open Source Software. 2024;9:5968. https://doi.org/10.21105/joss.05968.

3. Oxford Nanopore Technologies. Medaka. github.com/nanoporetech/medaka.

4. Bouras G. pypolca. 2024. https://github.com/gbouras13/pypolca.

5. Wick RR, Holt KE. Polypolish: Short-read polishing of long-read bacterial genome assemblies. PLOS Computational Biology. 2022;18:e1009802. https://doi.org/10.1371/journal.pcbi.1009802.

6. Low Wen Wen, Seddon Chloe, Beis Konstantinos, Frankel Gad. The Interaction of the F-Like Plasmid-Encoded TraN Isoforms with Their Cognate Outer Membrane Receptors. Journal of Bacteriology. 2023;205:e00061-23. https://doi.org/10.1128/jb.00061-23.

7. Thompson JD, Gibson TobyJ, Higgins DG. Multiple Sequence Alignment Using ClustalW and ClustalX. Current Protocols in Bioinformatics. 2003;00:2.3.1-2.3.22. https://doi.org/10.1002/0471250953.bi0203s00.

8. Tamura K, Stecher G, Kumar S. MEGA11: Molecular Evolutionary Genetics Analysis Version 11. Molecular Biology and Evolution. 2021;38:3022–7. https://doi.org/10.1093/molbev/msab120.

9. Letunic I, Bork P. Interactive Tree Of Life (iTOL): an online tool for phylogenetic tree display and annotation. Bioinformatics. 2007;23:127–8. https://doi.org/10.1093/bioinformatics/btl529.

10. Abramson J, Adler J, Dunger J, Evans R, Green T, Pritzel A, et al. Accurate structure prediction of biomolecular interactions with AlphaFold 3. Nature. 2024;630:493–500. https://doi.org/10.1038/s41586-024-07487-w.

11. Meng EC, Goddard TD, Pettersen EF, Couch GS, Pearson ZJ, Morris JH, et al. UCSF ChimeraX: Tools for structure building and analysis. Protein Science. 2023;32:e4792. https://doi.org/10.1002/pro.4792.
