# Supplementary figures and images for: Genomic and molecular characterisation of a KPC-producing Klebsiella pneumoniae clinical isolate resistant to meropenem-vaborbactam, imipenem-relebactam, and ceftazidime-avibactam
Source: BMC Genom Data. 2026 May 9;27:37. doi: 10.1186/s12863-026-01421-x (PMC13182041; doi:10.1186/s12863-026-01421-x)

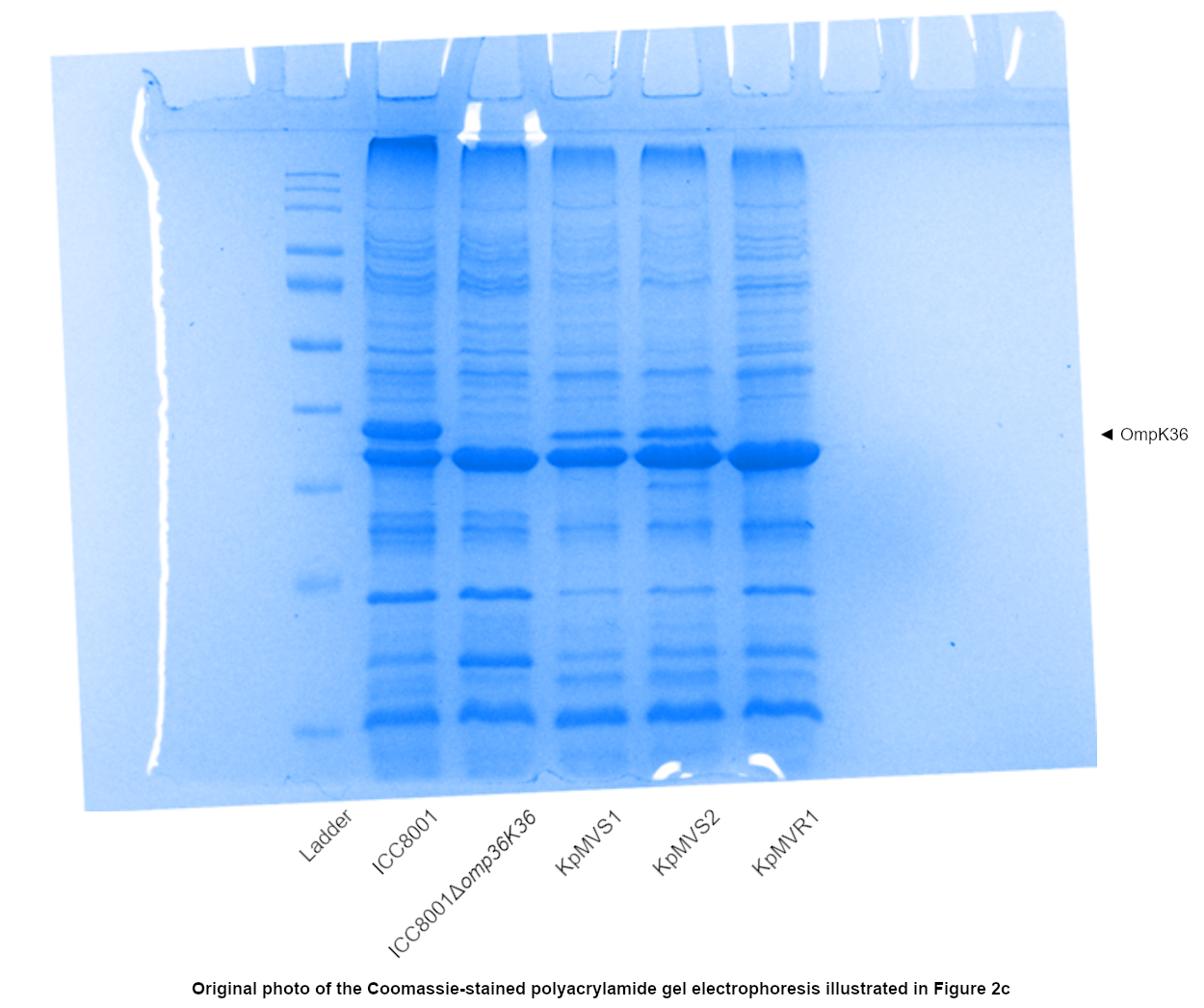

Supplement: Supplementary file 4 — Supplementary Material 4: Additional File 4 [file 12863_2026_1421_MOESM4_ESM.png]
